# The gene expression landscape of breast cancer is shaped by tumor protein p53 status and epithelial-mesenchymal transition

**DOI:** 10.1186/bcr3236

**Published:** 2012-07-27

**Authors:** Erik Fredlund, Johan Staaf, Juha K Rantala, Olli Kallioniemi, Åke Borg, Markus Ringnér

**Affiliations:** 1 Department of Oncology, Clinical Sciences and CREATE Health Centre for Translational Cancer Research, Lund University, Lund, Sweden; 2 Department of Biomedical Engineering and Knight Cancer Institute, Oregon Health and Science University, Portland, OR, USA; 3 Institute for Molecular Medicine Finland (FIMM), University of Helsinki, Helsinki, Finland

## Abstract

**Introduction:**

Gene expression data derived from clinical cancer specimens provide an opportunity to characterize cancer-specific transcriptional programs. Here, we present an analysis delineating a correlation-based gene expression landscape of breast cancer that identifies modules with strong associations to breast cancer-specific and general tumor biology.

**Methods:**

Modules of highly connected genes were extracted from a gene co-expression network that was constructed based on Pearson correlation, and module activities were then calculated using a pathway activity score. Functional annotations of modules were experimentally validated with an siRNA cell spot microarray system using the KPL-4 breast cancer cell line, and by using gene expression data from functional studies. Modules were derived using gene expression data representing 1,608 breast cancer samples and validated in data sets representing 971 independent breast cancer samples as well as 1,231 samples from other cancer forms.

**Results:**

The initial co-expression network analysis resulted in the characterization of eight tightly regulated gene modules. Cell cycle genes were divided into two transcriptional programs, and experimental validation using an siRNA screen showed different functional roles for these programs during proliferation. The division of the two programs was found to act as a marker for tumor protein p53 (*TP53*) gene status in luminal breast cancer, with the two programs being separated only in luminal tumors with functional p53 (encoded by *TP53*). Moreover, a module containing fibroblast and stroma-related genes was highly expressed in fibroblasts, but was also up-regulated by overexpression of epithelial-mesenchymal transition factors such as transforming growth factor beta 1 (TGF-beta1) and Snail in immortalized human mammary epithelial cells. Strikingly, the stroma transcriptional program related to less malignant tumors for luminal disease and aggressive lymph node positive disease among basal-like tumors.

**Conclusions:**

We have derived a robust gene expression landscape of breast cancer that reflects known subtypes as well as heterogeneity within these subtypes. By applying the modules to *TP53*-mutated samples we shed light on the biological consequences of non-functional p53 in otherwise low-proliferating luminal breast cancer. Furthermore, as in the case of the stroma module, we show that the biological and clinical interpretation of a set of co-regulated genes is subtype-dependent.

## Introduction

A large volume of breast cancer gene expression studies ultimately focus on deriving prognostic and predictive signatures, a few of which currently are considered for clinical use [1,2]. However, despite the availability of compilations of gene sets relating to specific cellular states or signaling pathways [3,4], the biological interpretation of large-scale gene expression data often comes in second place. Extracting cancer-specific signatures with biological relevance from genome-wide expression data could further our understanding of central tumor biological processes, their controlling factors and might help to delineate therapeutic considerations for cancer care, as well as the development of novel targeted therapies.

Gene expression profiling studies have substantiated that breast cancer can be divided into distinct disorders; and four main molecular subtypes have been identified: basal-like, Human Epidermal Growth Factor Receptor 2 (HER2)-enriched, luminal A and luminal B. Several different classifiers for molecular classification of clinical breast cancer specimens into these subtypes have been developed [5-7]. Although these classifiers, when applied to a group of patients, identify subtypes with similar survival there is considerable variation between classifiers in subtype assignments of individual samples [8]. Partly this variation reflects intra-subtype heterogeneity treated differently by different classifiers. One example of classification disagreement reflects differences in the separation into luminal A and B tumors, which mainly depends on proliferation-related genes with luminal B tumors displaying higher expression of such genes. The gene expression subtypes are reflected at the DNA copy number [9,10] and the DNA methylation levels [11,12]. However, some luminal A tumors have DNA copy number and methylation patterns similar to luminal B tumors, and patients with such luminal A tumors have worse outcome [10,12]. The above observations suggest a subset of luminal tumors that are clinically challenging despite a relatively low proliferative rate. Possibly these tumors share other features with the more aggressive luminal B subtype, except for high proliferation. Although the expression-based subtypes are related to different risks of recurrence and to clinical subtypes defined by measures of estrogen receptor (ER), HER2 and Ki-67 [5,7,13], it is clear that the subtypes are heterogeneous in terms of patient outcome. Consequently, we now begin to see a second generation of profiling studies aimed at stratifying molecular or clinical subtypes of breast cancer, based on the hypothesis that different prognostic or predictive markers will be needed for different subtypes [14]. Such studies have, for example, identified immune response signatures as having prognostic value in more challenging breast cancer subgroups, such as ER-negative, basal-like, HER2-positive or grade 3 tumors [15-17]. In addition, studies refining the subtypes and identifying additional subtypes are emerging [18,19].

In this study we have, using a computationally simple and biologically intuitive method, created a breast cancer-derived gene expression landscape with distinct modules reflecting central tumor biological themes. Our aim was to use a large set of tumors to define patterns of gene expression that can improve the understanding of heterogeneity within the breast cancer subtypes. Our results show the breast cancer landscape and its biological interpretation to be dependent on sample molecular traits and that these interpretations are conserved across multiple other cancer forms.

## Materials and methods

### Datasets

Gene expression modules were calculated from a dataset compiled from 10 independent studies, in total representing 1,608 breast cancer samples hybridized to Affymetrix HG-U133A arrays (U133A set; Additional file 1). The data were MAS5 normalized, mean centered across assays and samples were classified into molecular subtypes based on gene expression centroids from Hu *et al. *[6] as described [17]. Cross-hybridizing probes, defined as probes referring to more than one unique Entrez Gene ID or marked as cross-hybridizing by Affymetrix (x_at probes), were removed, and features were subsequently merged by calculating the mean expression of probes relating to the same Entrez Gene ID resulting in 12,208 gene-representative transcripts. Distant metastasis-free survival (DMFS) was not available for GSE3494 and GSE1456 and for these datasets relapse-free survival was used as a substitute for DMFS in survival analysis (Additional file 1). Clinical co-variates for the U133A set are described in Additional file 1. For validation of network modules a second gene expression breast cancer dataset representing 676 breast cancer samples was compiled from 12 independent studies performed on the Affymetrix HG-U133Plus2 platform (MAS5 normalized; Additional file 1). In addition, the NKI breast cancer dataset of 295 samples, representing an independent array technology, was used (Additional file 1). Additional datasets representing colon, ovarian, lung and bladder cancer, melanoma, diffuse large B-cell lymphoma and acute myeloid lymphoma are described in Additional file 2. For U133Plus2, data probes overlapping with the U133A platform were selected and expression data were merged based on Entrez Gene ID. Probe mapping between array platforms was done based on Entrez Gene IDs.

### Network construction and annotation

Prior to calculating correlations the data were filtered to remove non-varying genes. A standard deviation above 1 as cut-off criteria left the 3,824 (approximately 30%) most varying genes for further analyses. All pair-wise gene correlations were calculated for the 3,824 genes using a leave-one-out strategy: Pearson correlations between all possible gene pairs were calculated while excluding one dataset at a time; thus rendering a total of 10 correlation calculations. Only positive correlations above a set cut-off level across all these 10 calculations were used for further analyses; thereby, confounding factors inherent to single datasets were eliminated (Figure S1 in Additional file 3). Calculation of correlations between the 3,824 genes and a matrix with permuted class labels, repeated 1,000 times, gave a maximum random correlation of 0.14. Thus, a correlation above r = 0.14 could be considered significant (*P *= 0). Expression networks were created by connecting genes (nodes) by edges representing a minimum correlation across the 10 leave-one-out calculations above a set cut-off level, and then removing genes with less than 5 neighbors. To generate a gene expression landscape, we included genes from a network based on a correlation cut-off of 0.3, and visualized the network in Cytoscape using the pair-wise correlations as weights in a spring-embedded layout [20]. Next, each gene was placed in x-y space according to the r = 0.3 network layout and given a z-value based on the highest correlation cut-off at which it is in a network (using r = 0.3, 0.4, 0.5, 0.6 and 0.7 as cut-offs). Finally, the transcriptional landscape was visualized in R using the Krig, and Tps packages. Analysis using Spearman's rank correlation metric gave similar networks (data not shown). Modules within the created networks were mined for biological relevance using BINGO [21] and further text mining based analyses were performed using LitVan [22].

### Module expression

Module co-expression was evaluated by calculating the average pair-wise Pearson correlation between all genes for a module in a specific dataset. Co-expression values in validation data sets were compared to co-expression of 1,000 random gene sets of the same size (data not shown). In addition, the network average clustering co-efficient (NACC) was used as defined [23], that is, the fraction of the actual number of network connections within a defined gene module at a certain correlation cut-off level in relation to the maximum possible number of connections that could be obtained within that module. Module expression across samples was analyzed using a rank-based module activity score as previously described [24]. Relationships between module activity scores and sample annotations were analyzed using *t*-tests or ANOVA. For all survival analyses, patients were dichotomized on module expression above or below the module average and survival analyses were performed using the survival package in R. To control for dataset bias in survival analysis in the U133A set, robustness of results was evaluated in a leave-one-out analysis excluding one dataset at the time (data not shown). Correlations between network modules and individual genes were assayed using Spearman's rank correlations. All calculations presented were performed using PERL and R. All statistical tests were two-sided unless otherwise stated.

### Analysis of RNAi-based cell spot microarray data

KPL-4 breast cancer cells were seeded and grown on an array-based siRNA screening platform, and each siRNA was assayed for effects on Ki-67 immunohistochemistry staining intensity as previously described [25]. KPL-4 was a kind gift from Dr Junichi Kurebayashi, Department of Breast and Thyroid Surgery, Kawasaki Medical School, Japan [26]. The data from the siRNA screen are available in Additional file 4. Log-transformed raw intensities were used as Ki-67 staining intensities in all analyses. Group-wise effects on Ki-67 staining intensity for genes in the two proliferation modules were analyzed per module by comparing the mean module Ki-67 intensity to a random intensity distribution based on 10,000 sampled gene groups of the same size as the assayed module. Mean module intensities were for visualization purposes centered to the mean of the respective random intensity distribution. As a comparison, the same calculation was performed for the five siRNA controls present on the array platform.

## Results

### A breast cancer-specific transcriptional network

**Figure 1:**
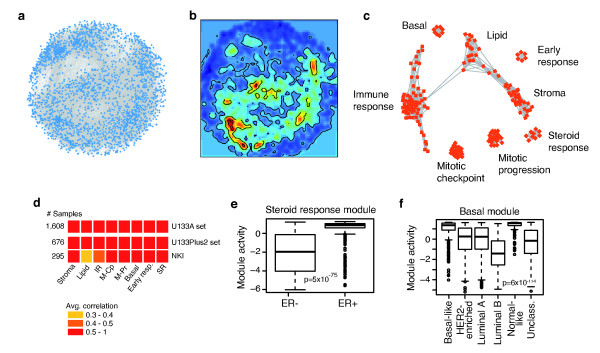
**A breast cancer gene expression network**. **(a) **Genes (represented as blue squares) with pair-wise gene expression correlations above 0.3 in a dataset representing 1,608 breast cancer samples were connected by edges and visualized using network graphics. Genes with less than five connecting edges were removed to extract a highly interconnected network. The network is complex and hard to interpret, even though all connections are statistically significant. **(b) **Although the network is dominated by regions of lower correlations (blue), there are regions in which genes are connected by higher correlations (red). **(c) **By restricting the analysis to genes with correlations above 0.6, a network of eight visually distinct modules reflecting the high correlation areas in (b) was extracted. In this way, the complex network in (a) could be reduced to a network with gene modules related to tumour biological themes. **(d) **Correlation-based modules were verified by assaying co-expression in independent breast cancer gene expression datasets. All pair-wise Pearson correlations between genes within modules were calculated across all samples for two additional breast cancer datasets representing 676 and 295 samples, respectively. The mean correlation for each module, as depicted by colored boxes, was used as a measure of module co-expression reproducibility. M-Pr, mitotic progression; M-Cp, mitotic checkpoint. **(e, f) **Module expression acts as surrogate markers for breast cancer molecular characteristics. (e) SR activity is high in ER-positive, but also in some ER-negative tumors. (f) Basal module activity is high in basal-like and normal-like tumors.

Many gene expression based studies of cancer have been hampered by small sample sets, but combining data from independent studies can potentially increase the power of such investigations [17,27-29]. We hypothesized that with a large number of samples, correlation in expression between genes becomes a powerful measure to identify core cancer-specific transcriptional programs. Therefore, we utilized a breast cancer gene expression dataset representing 1,608 samples combined from multiple sources (U133A set; Additional file 1) [17,30]. For this large sample size, even a very low correlation between genes was significant (Pearson's r > 0.14, *P *= 0, 1,000 permutations). However, when constructing gene expression networks by connecting correlated genes we observed that, even though the connections are statistically significant, extraction of distinct modules leading to biological interpretation of the network is difficult (Figure 1a). To address this issue, we generated a gene expression landscape by visualizing the network as a heat map to identify regions with higher correlations (Figure 1b). A common concern with high throughput data is batch effects [31]. Importantly, we found that the influence of data source on results decreased with increasing correlations and became negligible at r > 0.6 (Figure S1 in Additional file 3). A network derived from correlations larger than 0.6 contained 187 genes with 1,272 connections distributed in eight visually distinct modules (Figure 1c, Additional file 5). We validated the co-expression of the modules in two independent breast cancer datasets representing 676 and 295 samples, respectively (Figure 1d, Additional file 1). Surprisingly, when testing in excess of 5,000 functionally annotated sets [4] none reached the level of co-expression observed for our modules (Figure S2 in Additional file 3), supporting the value of identifying cancer-specific transcriptional programs.

Based on published associations to breast cancer-specific tumor biology, a steroid response module (SR), a basal breast cancer module (basal), and a module containing genes (for example, *FOS *and *EGR1*) [32] related to early response to growth factor or serum stimulation (early response) were identified (Figure 1c, Additional file 5). Furthermore, one module (lipid) was representative of adipocytes, containing markers of terminal differentiation along that lineage (for example, *ADIPOQ, PLIN*) [33]. Additional mapping of module genes to known pathways and ontology terms suggested the remaining four modules to be associated with the cell cycle (mitotic checkpoint and mitotic progression), immune response (IR) and extracellular matrix-related processes (stroma) (Figure 1c, Additional files 5 and 6). Hence, gene expression landscape analysis is an intuitive approach for identifying biologically relevant transcriptional programs.

### Modules are markers for tumor subtype-specific processes

In order to relate module gene expression to clinical parameters and breast cancer subgroups, a rank-based module activity score [24] was calculated for each of the eight modules in each breast cancer sample (Figure S3 in Additional file 3). The SR module contained known ER-status-related genes, such as *GATA3*, *CA12, XBP1 *and *FOXA1 *[34-37], and by correlation to module activity scores the expression of ER-alpha (*ESR1*) and the progesterone receptor (*PGR*) were strongly associated to this module (Spearman's rho = 0.65 and 0.50, respectively). The activity scores for the SR module showed a distinct bimodal distribution with a high activity in ER-positive as compared to ER-negative cases (*P *= 5*10^-75^, *t*-test) (Figure 1e). Intuitively, one would expect the SR module to be specific for ER-positive tumors; however, some ER-negative cases also showed high SR activity (Figure 1e). A comparatively high expression of the androgen receptor (*AR*) within this subgroup of ER-negative samples (*P *= 4*10^-44^, *t*-test; Figure S4 in Additional file 3) suggested these cases to be of the apocrine breast cancer subtype [38]. Thus, high activity of the SR module can act as a functional indicator for a general steroid response.

The basal module, containing known basal cell keratins *KRT5*, *KRT14 *and *KRT17 *[39], showed a subtype-specific bimodal activity score distribution with high module activity in the basal-like and normal-like subtypes (*P *= 6*10^-114^, ANOVA) (Figure 1f). The IR module showed the highest activity in the basal-like and HER2-enriched subtypes (Figure S5 in Additional file 3), and within those subtypes high IR module activity was significantly associated with more favorable prognosis (*P *= 0.005 and *P *= 0.003, respectively, log-rank tests) as previously reported [17,27].

### Cell cycle genes are separated into two modules dependent on TP53 status

Our gene expression landscape showed two distinct modules (mitotic checkpoint and mitotic progression) that both contained genes related to central mitotic processes (Figure 1c). These two cell cycle modules were difficult to differentiate with respect to function. Genes in both modules were in general annotated to similar gene ontology terms and, in particular, the majority of genes in both modules were annotated to the term M-phase (Additional file 6). However, when focusing on the differences between the two modules, we observed that in the mitotic checkpoint module there were four genes annotated to spindle checkpoint (*MAD2L1*, *TTK*, *BIRC5*, *CENPE*) and six genes annotated to regulation of cell cycle (*CKS2*, *MAD2L1*, *TTK*, *BIRC5*, *CENPE*, *DLGAP5*), whereas no genes were annotated to these terms in the other module. However, in the mitotic progression module, six genes were annotated to the microtubule cellular compartment (*KIF4A*, *KIF15*, *KIF18A*, *KIF18B*, *KIF20*, *NUSAP1*, and *PRC1*, of which five were annotated to microtubule-based movement), and six genes were annotated to DNA binding (*E2F8*, *HJURP*, *EXO1*, *ERCC6L*, *KIF15*, *KIF4A*, *NUSAP1*), whereas no genes in the mitotic checkpoint module were annotated to these categories. These differences indicated that one module is more related to regulation of the M-phase and the mitotic checkpoint, while the other module seemed more related to carrying out the M-phase. Literature mining [22] corroborated these differences (Figure S6 in Additional file 3).

**Figure 2:**
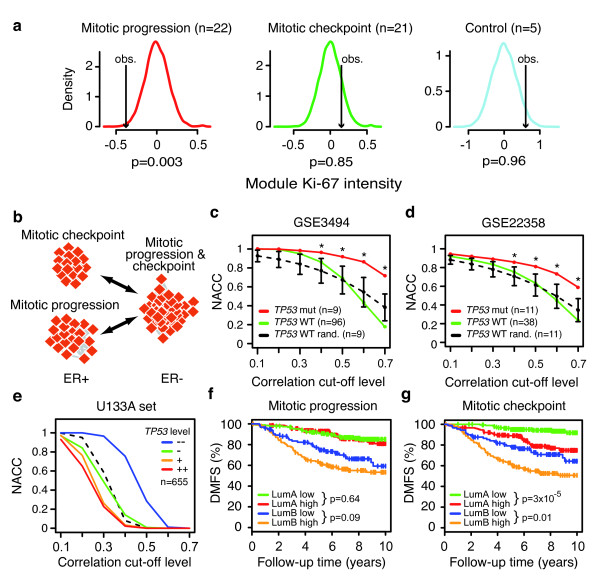
**Separation of cell cycle genes into two modules is dependent on *TP53 *status**. **(a) **Module genes were assayed for effects on proliferation in the KPL-4 breast cancer cell line using an RNAi-based cell spot microarray system. Knockdown of genes in the mitotic progression module significantly inhibited cell proliferation as assayed using Ki-67 staining intensity (*P *= 0.003, left panel), whereas knockdown of genes in the mitotic checkpoint module did not show any significant effects (*P *= 0.85, center panel). A group of non-specific control siRNAs showed that the majority of genes in the assayed siRNA library abrogate cellular proliferation (right panel). Module effects on KPL-4 proliferation was estimated by comparing the observed mean Ki-67 intensity for the module genes (black arrows) and compared to background Ki-67 distributions (density curves) based on 10,000 random groups of the same size as the assayed module. *P*-values are one-sided. **(b) **The mitotic progression and checkpoint modules are separated in ER-positive breast cancer, but interconnected as a single module in ER-negative breast cancer. **(c, d) **The separation of the mitotic progression and checkpoint modules relate to sample *TP53 *mutation status. Interconnection between the mitotic progression and checkpoint modules were assayed using the NACC at increasing cut-off correlation levels in luminal A and luminal B samples. NACC was calculated in luminal samples with known *TP53 *mutation status from the (c) GSE3494 and (d) GSE22358 breast cancer datasets. *TP53 *wildtype (WT) samples showed a clear separation between the mitotic progression and checkpoint modules at increasing correlation cut-off levels (green lines). However, in *TP53*-mutated samples modules remained interconnected at higher levels of correlation (red lines). The NACC for *TP53*-mutated samples was compared to 10,000 random selections of the same number of *TP53 *WT samples (black dashed lines) and stars denote permutation-based p-values below 0.05. Error bars represent standard deviations. **(e) **Luminal samples from the U133A set were divided into quartile groups based on *TP53 *expression and NACC between mitotic progression and checkpoint modules were calculated within these groups. Decreasing *TP53 *expression correlated to higher level of interconnection between the mitotic progression and checkpoint modules with the highest *TP53 *expression quartile samples showing a distinctly higher module interconnection than the lowest quartile samples. As reference the NACC for all luminal samples is shown (black dotted line). **(f) **Dichotomizing breast cancer patients of either luminal A (LumA) or luminal B (LumB) subtype on mitotic progression module activity did not add prognostic information (*P *= 0.6 and *P *= 0.09, log-rank tests) using DMFS as endpoint, **(g) **while an above mean activity of the mitotic checkpoint module identified groups within both luminal A and luminal B tumors with worse prognosis (luminal A *P *= 3*10^-5^, luminal B *P *= 0.01, log-rank tests).

To experimentally investigate the functional differences of the two mitotic modules suggested by our computational analyses, we utilized a high-throughput RNAi-based cell spot microarray screening method [25]. KPL-4 breast cancer cells were reverse transfected with a library of siRNAs targeting 5,760 genes and Ki-67 intensity was assayed as a marker for cellular proliferation [25]. By combining Ki-67 intensity data for genes in the mitotic checkpoint and progression modules separately, we could investigate module level effects on proliferation. As expected, knockdown of the mitotic progression module genes resulted in significantly lowered Ki-67 staining (Figure 2a, left) as compared to a group of unspecific control siRNAs (Figure 2a, right), suggesting that the mitotic progression genes are pivotal for progression through the cell cycle. However, knockdown of the mitotic checkpoint module genes did not result in lowered Ki-67 intensity (Figure 2a, middle), suggesting that knockdown of the mitotic checkpoint genes does not hinder mitotic progression. These results support our annotation of the modules to separate cell cycle processes and to denote them mitotic progression and mitotic checkpoint, respectively.

Elevated expression of mitotic checkpoint genes has been associated with chromosomal instability in breast cancer cells [40,41], and the mitotic checkpoint module genes showed a considerable overlap with a signature for chromosome instability in tumors [42]. Moreover, high expression of *TTK *(*MPS1*) in our mitotic checkpoint module has been reported to promote aneuploidy in breast cancer [43]. Since the mitotic checkpoint and progression genes have been shown to be co-expressed in normal tissue [44], we suspected that they were separated in breast cancer because a subgroup of tumors challenged by chromosomal instability contained cells with a halted progression through the cell cycle [45]. To identify such tumors we investigated correlation between the mitotic checkpoint and progression modules within subgroups of breast cancer [23]. While the modules remained distinct in ER-positive samples as well as the luminal A and B subtypes, they were more interconnected in ER-negative samples and the basal-like subtype (Figure 2b, Figure S7a in Additional file 3).

Cells with a stressed mitotic checkpoint accumulate genomic aberrations [40,41], but are subject to the p53-dependent G_1 _post-mitotic checkpoint, which acts as an additional barrier against proliferation of aberrant cells [46]. Furthermore, proliferation of aneuploid daughter cells is strongly linked to p53 status [47]. Therefore, we investigated whether the separation of proliferation genes into two distinct modules in luminal tumors was related to p53 functional status. To this aim, we calculated the network average clustering co-efficient (NACC) between the mitotic checkpoint and progression modules in luminal samples with known *TP53 *status [48]. Indeed, we observed that while the mitotic checkpoint and progression modules were separated in *TP53*-wildtype samples they were connected in *TP53*-mutated samples (*P *< 0.05) (Figure 2c). Importantly, this finding was validated in an independent dataset (Figure 2d) [49]. In addition, low levels of *TP53 *gene expression correlated to increased interconnectivity between the two mitotic modules in luminal samples (Figure 2e), and *TP53*-mutated luminal samples showed elevated activity of the mitotic checkpoint and progression modules (*P *= 5*10^-4 ^and *P *= 9*10^-4^, *t*-tests). Furthermore, the vast majority of basal-like tumors has dysfunctional p53 and displays high chromosomal instability, and the two mitotic modules were not separated in these tumors (Figure S7a in Additional file 3). Together, these analyses suggest a subgroup of genomically unstable luminal tumors with proliferation hindered by functional p53.

To investigate whether elevated activity of the mitotic checkpoint and progression transcriptional programs translated into disease aggressiveness, we performed survival analyses within the luminal A and B subtypes separately. The mitotic progression module only showed marginal prognostic capability within these subtypes (Figure 2f). However, high activity of the mitotic checkpoint module correlated significantly to unfavorable prognosis in both luminal subgroups (luminal A *P *= 3*10^-5^, luminal B *P *= 0.01, log-rank tests) (Figure 2g). Thus, genes in the mitotic checkpoint module relate to a more aggressive disease phenotype within the otherwise low proliferating luminal A tumors, but also within the more highly proliferating luminal B tumors. Correspondingly, the mitotic checkpoint module was predictive for distant metastasis free survival (DMFS) within both grade 1 and grade 2 tumors (*P *= 0.007 and *P *= 1*10^-4^, respectively, log-rank tests), whereas the mitotic progression module only was predictive for grade 2 tumors (*P *= 0.001, log-rank test) (Figure S7b in Additional file 3).

### The stroma module is related to epithelial-mesenchymal transition

**Figure 3:**
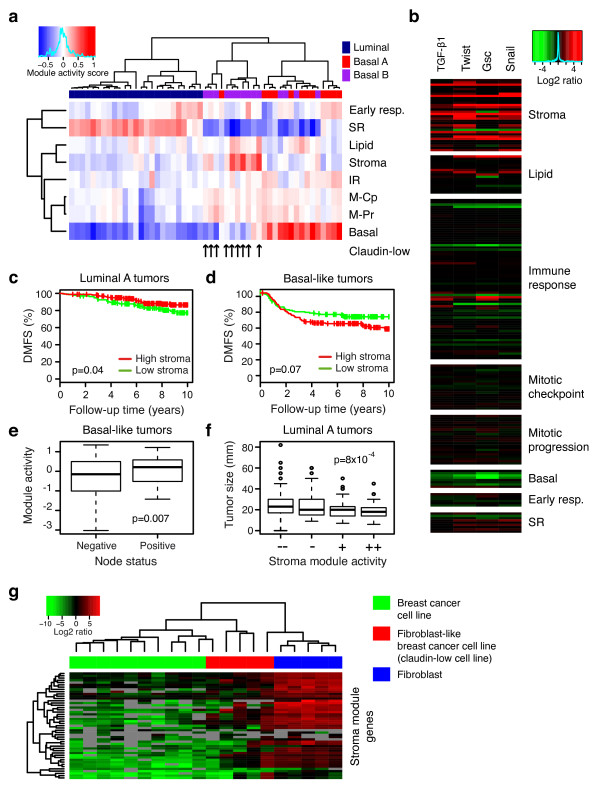
**The stroma module represents mesenchymal cell characteristics**. **(a) **Hierarchical clustering of module activity scores, calculated in data representing 51 breast cancer cell lines, showed separation into the main cell line types: luminal, basal A and basal B [50]. Black arrows denote cell lines characterized as representing a claudin-low phenotype [51]. M-Pr, mitotic progression; M-Cp, mitotic checkpoint. **(b) **Expression of EMT-inducing factors increases expression of genes from the stroma module. Data for the 187 module genes from a dataset representing overexpression of TGF-beta1, Twist, Gsc or Snail in immortalized breast fibroblasts were visualized using heatmaps. Data are shown as fold changes in relation to mock transfection control. **(c) **A high stroma module activity score correlates to a more favorable prognosis in patients of the luminal A subtype (*P *= 0.04, log-rank test), whereas **(d) **an opposite trend was observed for patients with tumors of the basal-like subtype (*P *= 0.07, log-rank test). Patients were dichotomized based on a stroma module activity score above or below mean within each subtype. **(e) **Within the basal-like classified patients a high stroma module activity score correlated to node-positive disease (*P *= 0.007, *t*-test). **(f) **Within the luminal A classified patients a higher stroma module activity score, quantized into four groups, correlated to a smaller tumor size (*P *= 8*10^-4^, ANOVA). **(g) **Hierarchical clustering of primary breast fibroblasts, fibroblast-like (claudin-low) breast cancer cell lines, and breast cancer cell lines, based on expression of genes in the stroma module. Data from GSE13915 [54].

Cell lines have previously been shown to emulate molecular breast cancer subtypes, especially with regard to basal-like and luminal disease [50]. We calculated activity scores for the eight modules in gene expression data representing 51 breast cancer cell lines [50]. Hierarchical clustering of the module activity scores clearly separated the cell lines into luminal and basal groups (Figure 3a). The luminal cell lines showed exclusively high activity of the SR module, whereas the basal A and B cell lines showed comparatively higher activity of the proliferation-related modules (Figure 3a). Furthermore, low activity of the basal module together with high activity of the stroma module gave a cluster highly enriched for the basal B classified cell lines and cell lines recently defined as claudin-low (Figure 3a), suggesting that high activity of the stroma module relates to a more mesenchymal cell phenotype [51]. The stroma module was enriched for genes related to matrix remodeling processes (for example, *VCAN*, *FBN1*, *DCN*, *MMP2*; Additional file 5) and literature mining suggested an association to TGF-beta signaling (Figure S8 in Additional file 3), a pathway known to be involved in epithelial-mesenchymal transition (EMT) [52]. In order to further investigate a relationship between the stroma module and EMT, we used microarray data derived from induced expression of known EMT-inducing factors SNAI1, TWIST, GSC or TGF-beta1 in an immortalized human mammary epithelial cell system [53]. All of the four EMT-inducing factors clearly up-regulated genes from the stroma module, while, interestingly, genes in the basal module showed reduced expression (Figure 3b). In the clinical breast cancer data we observed a similar expression pattern of the stroma module as for a previously reported EMT-signature [53], that is, higher expression in luminal A as compared to basal-like tumors (Figure S3 in Additional file 3). However, for luminal A tumors high stroma module activity correlated to more favorable prognosis (*P *= 0.04, log-rank test) (Figure 3c), whereas the opposite trend was observed for basal-like tumors (*P *= 0.07, log-rank test) (Figure 3d). Furthermore, stroma module activity was higher in node positive as compared to node negative patients of the basal-like subtype (basal-like *P *= 0.007, luminal A *P *= 0.7, *t*-tests) (Figure 3e). In contrast, for luminal A samples high stroma module activity reflected small tumor size (*P *= 8*10^-4^, basal-like *P *= 1, ANOVA) (Figure 3f), indicative of less aggressive disease. While a majority of the genes in the stroma module were regulated by EMT-inducing factors, many of the stroma genes are also well known fibroblast markers. Therefore, we investigated the expression of the stroma module genes in data representing primary breast fibroblasts [54]. Indeed, several of the stroma genes were also highly expressed in primary breast fibroblasts (Figure 3g). In conclusion, due to the heterogeneity of breast cancer, a transcriptional program may reflect different processes and have opposite biological effects in different breast cancer subtypes. Thus, the interpretation of a gene expression signature is highly dependent on subtypes, and both intra- and intertumoral heterogeneity should be considered.

### Breast cancer modules are co-expressed in other cancer forms

**Figure 4:**
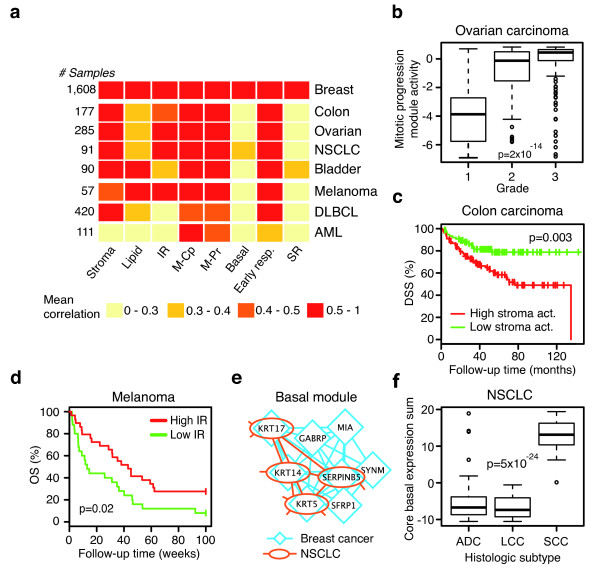
**The breast cancer-derived gene expression modules are preserved across several cancer forms**. **(a) **The breast cancer gene expression modules were assayed for co-expression in data representing seven other cancer forms by calculating the average pair-wise Pearson correlation for genes within each module separately. All observed correlations were significant as compared to a random average pair-wise correlations based on 1,000 permutations (data not shown) M-Pr, mitotic progression; M-Cp, mitotic checkpoint. **(b) **A high activity score of the mitotic progression module correlated to increasing grade in an ovarian carcinoma dataset (*n *= 285, *P *= 2 × 10^-14^, ANOVA). **(c) **An above mean expression of genes in the stroma module correlates to decreased disease-specific survival (DSS) in a colon carcinoma dataset. (*n *= 177, *P *= 0.003, log-rank test). **(d) **A high immune response (IR) module activity correlated to favorable overall survival (OS) in a dataset representing 57 stage IV melanomas (*P *= 0.02, log-rank test). **(e) **Calculation of pair-wise Pearson correlations in an NSCLC dataset for genes in the breast cancer basal module (blue network) revealed that only a subset of these genes were correlated in NSCLC (red network). A core basal gene expression module (*n *= 5) was derived from genes with conserved correlations in both breast and lung cancer data (red network). **(f) **A high expression sum for the core basal module acted as a marker for squamous cell lung carcinoma (SCC) compared to the other NSCLC morphological types adenocarcinoma (ADC) and large cell carcinoma (LCC) (*P *= 5*10^-24^, ANOVA).

Since several of the identified gene expression modules represented processes of broader influence on tumor progression, we assayed module co-expression in seven different cancer forms, including four carcinomas (colon, non-small cell lung carcinoma (NSCLC), ovarian and bladder), stage IV malignant melanoma, diffuse large B-cell lymphoma (DLBCL) and acute myeloid leukemia (AML) (Additional file 2). As expected, the proliferation-related modules were co-expressed across all assayed cancer forms (Figure 4a) and, in line with this, activity scores for the two mitotic modules showed a significant correlation to increasing tumor grade in ovarian carcinoma (*P *= 2*10^-14 ^and 5*10^-15^, respectively. ANOVA) (Figure 4b). However, some modules were co-expressed only in certain cancer forms. For instance, the SR module was found only in breast and bladder cancer. Interestingly, it has been reported that a subgroup of bladder cancer have high AR expression [55], suggesting a gene expression scenario similar to AR-positive apocrine breast cancer. The breast cancer-derived stroma module was co-regulated in several of the assayed tumor datasets, including colon carcinoma (Figure 4a). As EMT is known to be involved in the canonical colorectal adenoma-carcinoma sequence [52], we tested whether activity of this module related to colon carcinoma patient outcome. Indeed, patients with high activity of the stroma module showed poorer disease-specific survival than patients with low stroma activity (*P *= 0.003, log-rank test; Figure 4c). Moreover, stroma module activity was independent of tumor stage or grade in this dataset (*P *= 7*10^-4^, HR 3.0, 95% CI 1.6 to 5.6, Cox regression). A previous report has shown that a gene expression signature relating to tumor infiltrating lymphocytes is prognostic in advanced melanoma [56]. Correspondingly, the high activity of our IR module, mainly containing genes related to activated cytotoxic T-lymphocytes (Additional files 5 and 6), correlated to more favorable prognosis in patients with stage IV melanoma (Figure 4d). These analyses show that not only are certain gene expression modules conserved across several cancer forms, but also suggest that the biology reflected by these transcriptional programs is generally descriptive for tumor biology and clinical outcome.

Despite overlap with known markers for squamous cell morphology (for example, *KRT5*, *KRT14*, *KRT17*) [39,57], the breast cancer basal module did not show strong co-expression in any of the other cancer forms. To investigate this, we created a gene expression network originating from the genes in the breast cancer-specific basal module using data representing 91 NSCLC [58]. We observed that while a core set of genes from the breast cancer module retained their high correlations, a large proportion of the gene-gene correlations were not present in the NSCLC data (Figure 4e). Using this core basal module (Figure 4e), we calculated expression sums for these genes in the NSCLC data and compared to tumor morphological type. Squamous cell lung carcinomas showed a strikingly higher expression of genes in the core basal module as compared to both adenocarcinomas and large cell carcinomas (*P *= 5*10^-24^, ANOVA) (Figure 4f). Moreover, the core basal module showed higher co-expression in colon, ovarian and bladder cancer, as well as in DLBCL, suggesting this gene expression motif is highly conserved in cancers encompassing subtypes with basal or squamous morphology (Figure S9 in Additional file 3). These results show that a transcriptional program that is common to several cancer types contain a core set of genes that are correlated to additional genes in a cancer-specific manner. This may reflect that conserved cancer processes are regulated by distinct spectra of aberrations in different cancer forms.

## Discussion

In this study we uncovered a breast cancer gene expression landscape with eight gene modules reflecting biologically relevant transcriptional programs conserved in other cancer forms. At least three of these likely relate to infiltration or presence of stromal or immune cells in the macro-dissected tissue used for the microarray experiments. However, transcriptional programs can reflect different processes and have different association with disease aggressiveness depending on context. In concordance with previous reports, we find that high steroid response can reflect signaling by either ER or AR [38], and that high expression of genes relating to an immune response correlate to favorable outcome in ER-negative or HER2-enriched disease only [17,27,28]. We now report a gene expression module containing stroma-related genes that were highly expressed in normal fibroblasts. For luminal A tumors, high activity of this stroma module more likely reflected infiltrating fibroblasts or the presence of normal tissue, which in our data corresponded to small tumor size and favorable patient outcome. Among basal tumors we could see the opposite trend, probably related to EMT of the cancer cells as a strong up-regulation of the stroma module genes could be seen when inducing EMT in immortalized epithelial cells [53]. However, no EMT master regulator genes were present in this gene expression module. EMT has previously been associated with basal-like breast cancers [59] and we extend these results by showing that our EMT-induced stroma module correlates to disseminated and aggressive disease specifically within this subtype. Interestingly, this phenotype also corresponds to the claudin-low subtype [51] with high expression of EMT markers and low expression of basal markers, although our analyses did not extract a claudin-low module. Together, these results suggest that two sources of major heterogeneity within basal-like tumors are related to immune-response and EMT-related processes. By basing our modules on such a large number of tumors, a possibility is to evaluate them as robust biomarkers; not only as prognostic markers for breast cancer and other cancer forms as shown here, but also as predictive markers of treatment response. Indeed, a recent study has shown that expression of certain EMT-associated genes is more pronounced in post-treatment breast cancer samples [60].

Defective cell cycle checkpoints affect cell cycle phase lengths and the fraction of cells arrested in different phases, which can be reflected in gene expression profiles [45]. Our observation of separate cell cycle modules in luminal breast cancers dependent on *TP53 *status suggests that luminal tumors can be further stratified independently of proliferation, and supports a picture in which defective cell cycle checkpoints do not always correspond to high proliferative rates in breast cancers [10]. Correspondingly, the mitotic checkpoint module stratifies histological grade 1 and luminal A tumors as well as histological grade 2 and luminal B tumors into groups with differential prognosis (Figure S7 in Additional file 3), which may also translate into differential response to chemotherapy. Hence, these results add information beyond proliferation-associated signatures, such as the Genomic Grade Index [61], but also beyond published *TP53 *mutation and chromosomal instability signatures so far mainly focused on basal-like tumors as these characteristics are significantly more frequent in ER-negative tumors [48,62]. Coutant *et al. *have recently identified distinct p53 gene signatures in ER-positive and ER-negative breast cancers and, interestingly, the ER-positive p53 gene signature was predictive of response to both adjuvant chemotherapy and tamoxifen [63]. Our findings suggest that a detailed analysis of cell cycle genes may provide a better understanding of the inconsistencies between proliferation-based classifiers of luminal breast cancer [8] and open the door for improved stratification of these patients.

Our study exemplifies that for large sample sets correlation in expression is a powerful measure to identify core gene modules that can be more easily associated with specific biological and genetic traits. Furthermore, we show that gene expression modules can act as robust biomarkers not only for genetic traits [64], but also for differential composition of the tumor microenvironment. As the number of available tumor expression profiles increases, the broad view presented here should be extended by identifying additional transcriptional programs relevant only within specific patient cohorts.

## Conclusions

The presented results highlight that the biological and clinical interpretations of gene expression based transcriptional programs are subtype-dependent, and that both intra- and intertumoral heterogeneity should be considered for realizing the full potential of omics-type tumor data. Moreover, using a novel approach we show that differences in correlation between functional gene modules can be used as gene expression-based signatures for genetic aberrations.

## Abbreviations

AML: acute myeloid leukemia; DLBCL: diffuse large B-cell lymphoma; DMFS: distant metastasis-free survival; DSS: disease-specific survival; EMT: epithelial-mesenchymal transition; ER: estrogen receptor; HER2: Human Epidermal Growth Factor Receptor 2; IR: immune response; NACC: network average clustering coefficient; NSCLC: non-small cell lung carcinoma; OS: overall survival; SR: steroid response; siRNA: small interfering RNA; TGF-beta1: transforming growth factor beta 1; TP53: tumor protein p53.

## Competing interests

The authors declare that they have no competing interests.

## Authors' contributions

EF and MR conceived of the study, designed the analyses and wrote the manuscript. EF performed the analyses. JS contributed to data analysis. JKR and OP performed RNAi-based cell spot microarray screenings. ÅB contributed to data interpretation. All authors read and approved the final manuscript.

## Supplementary Material

Additional file 1 **Breast cancer gene expression data sets**. An Excel table with description of all breast cancer gene expression data sets. Click here for file

Additional file 2 **Gene expression data sets from other cancer forms**. An Excel table with description of gene expression data sets for other cancer forms. Click here for file

Additional file 3 **Supplementary figures**. A pdf file containing all supplementary figures with legends (Figure S1-S9). Click here for file

Additional file 4 **Ki-67 data from siRNA screen**. An Excel file with Ki-67 staining intensity data from an siRNA screen in KPL-4 breast cancer cells, as well as further description of data and methodology. Click here for file

Additional file 5 **Network module genes**. An Excel table with all genes in the gene expression network modules. Click here for file

Additional file 6 **Gene ontology analysis**. An Excel table with gene ontology biological processes enriched in the gene expression network modules. Click here for file
