# Supplementary material for: The gene expression landscape of breast cancer is shaped by tumor protein p53 status and epithelial-mesenchymal transition
Source: Breast Cancer Res. 2012 Jul 27;14(4):R113. doi: 10.1186/bcr3236 (PMC3680939; doi:10.1186/bcr3236)

# Supplementary Figure Legends

**Supplementary Figure 1. Multidimensional scaling to investigate batch effects.** Multidimensional scalings into two dimensions are shown for the 1,608 samples in the U133A-set. The multidimensional scalings are restricted to genes in gene expression networks. Samples are colored according to dataset. For networks based on lower correlation cut-offs to connect genes (e.g. 0.3) there is an influence from data source, but for higher cut-offs (e.g. 0.6) the influence from data source has decreased.

**Supplementary Figure 2. Co-expression of genes in annotated gene sets.** The correlation in gene expression of genes in more than 5,000 annotated gene sets from MSigDB was investigated in the U133A-set. **(a)** No gene set had an average pairwise Pearson correlation larger than 0.6 and only 56 gene sets had an average correlation above 0.3. The results were not related to size of the gene set. **(b)** The 56 gene sets with correlation above 0.3 were mainly related to cell cycle, immune response and ribosomes. **(c)** Of the gene sets showing some co-expression a large proportion were specifically derived from partly breast cancer-based co-expression neighborhood analyses (CGN).

**Supplementary Figure 3. Module activity in breast cancer molecular subtypes.** The module activity score for each module is shown for samples in the U133A-set stratified by molecular subtypes.

**Supplementary Figure 4. *AR* expression in ER-negative samples.** The expression of *AR* is significantly higher in ER-negative samples with high steroid response module activity than in ER-negative samples with low steroid response module activity.

**Supplementary Figure 5. The immune response module in breast cancer. (a)** The immune response (IR) module displays higher activity in ER-negative than in ER-positive tumors. **(b)** The IR module displays higher activity in basal-like and HER2-enriched samples than in samples of other subtypes. **(c)** A below mean activity of the IR module identifies basal-like tumors with poor prognosis. **(d)** A below mean activity of the IR module identifies poor prognosis HER2-enriched tumors. **(e)** IR module activity does not stratify luminal A tumors in groups with different prognosis.

**Supplementary Figure 6. Functional annotation analysis of the two cell-cycle related modules.** Genes in the mitotic progression (green) and mitotic checkpoint (yellow) modules were subjected to literature mining for functional annotations using Litvan literature mining.

**Supplementary Figure 7. The mitotic progression and checkpoint modules in breast cancers stratified by molecular and clinical subgroups. (a)** The network

average clustering coefficient (NACC) for the mitotic progression and checkpoint modules is shown for different cut-offs in correlation used to connect genes in networks. At higher correlation cut-offs the mitotic progression and checkpoint modules are separated in ER-positive but not in ER-negative samples (left). The mitotic progression and checkpoint modules are more interconnected in basal-like tumors than in tumors of other subtypes (right). **(b)** High activity of the mitotic checkpoint module correlated to unfavorable prognosis in both grade 1 and grade 2 tumors but not in grade 3 tumors (left), whereas high activity of the mitotic progression module only correlated to unfavorable prognosis in grade 2 tumors (right). M-Pr, mitotic progression; M-Cp, mitotic checkpoint.

**Supplementary Figure 8. Functional annotation analysis of the stroma module.** Genes in the stroma module were subjected to literature mining for functional annotations using Litvan literature mining.

**Supplementary Figure 9. Co-expression of basal module in different cancer types.** The co-expression of the basal module derived from breast cancer is compared to the co-expression of the core basal module, which consists of genes in the breast cancer derived basal module also co-expressed in NSCLC. The core basal module generally showed higher co-expression for other cancer types.

Supplementary Figure 1

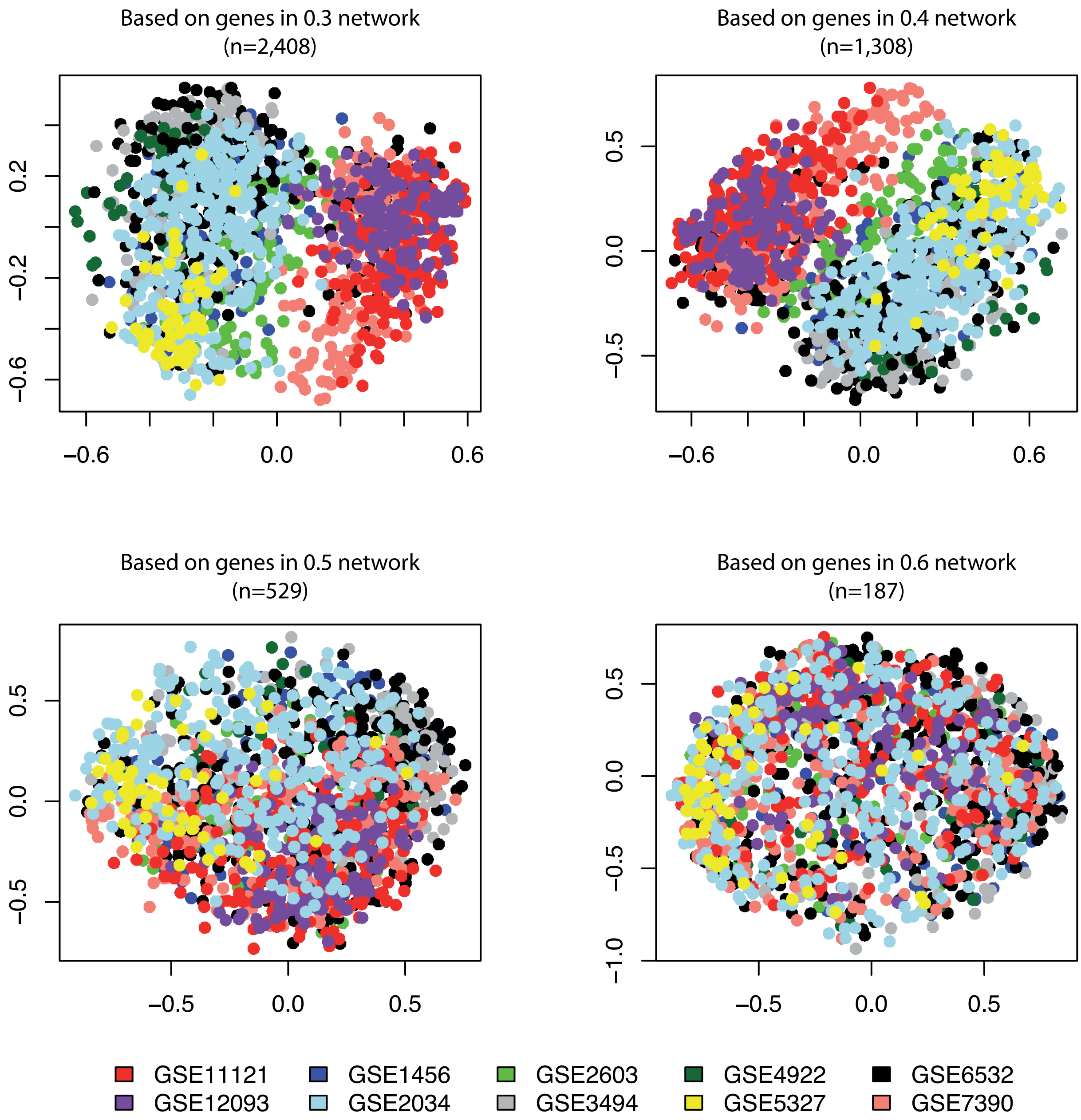

Supplementary Figure 2

**a**

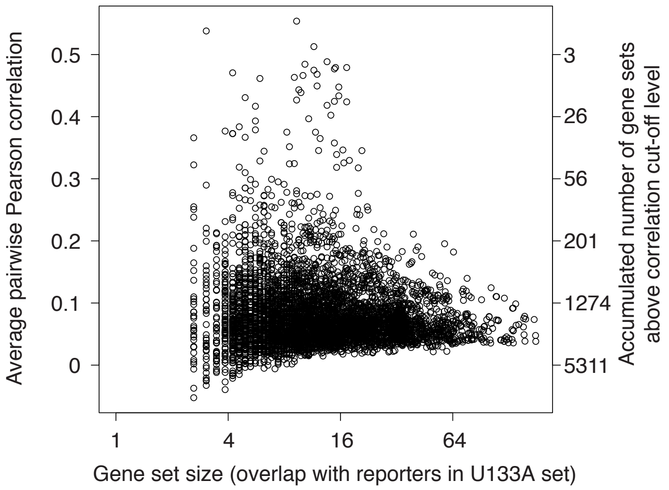


**b**

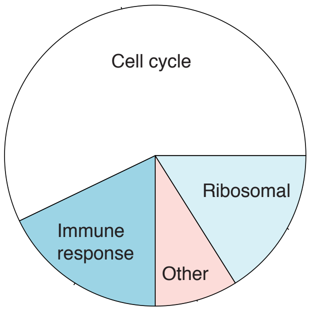


Main functional categories of GSEA gene sets with an average Pearson correlation above 0.3 (n=56)

**c**

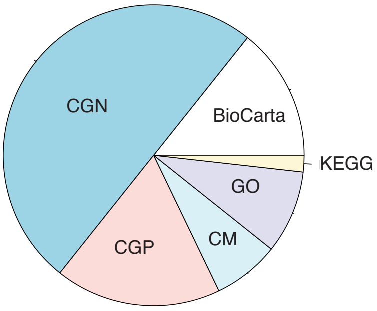


Source of GSEA gene sets with an average Pearson correlation above 0.3 (n=56)

Supplementary Figure 3

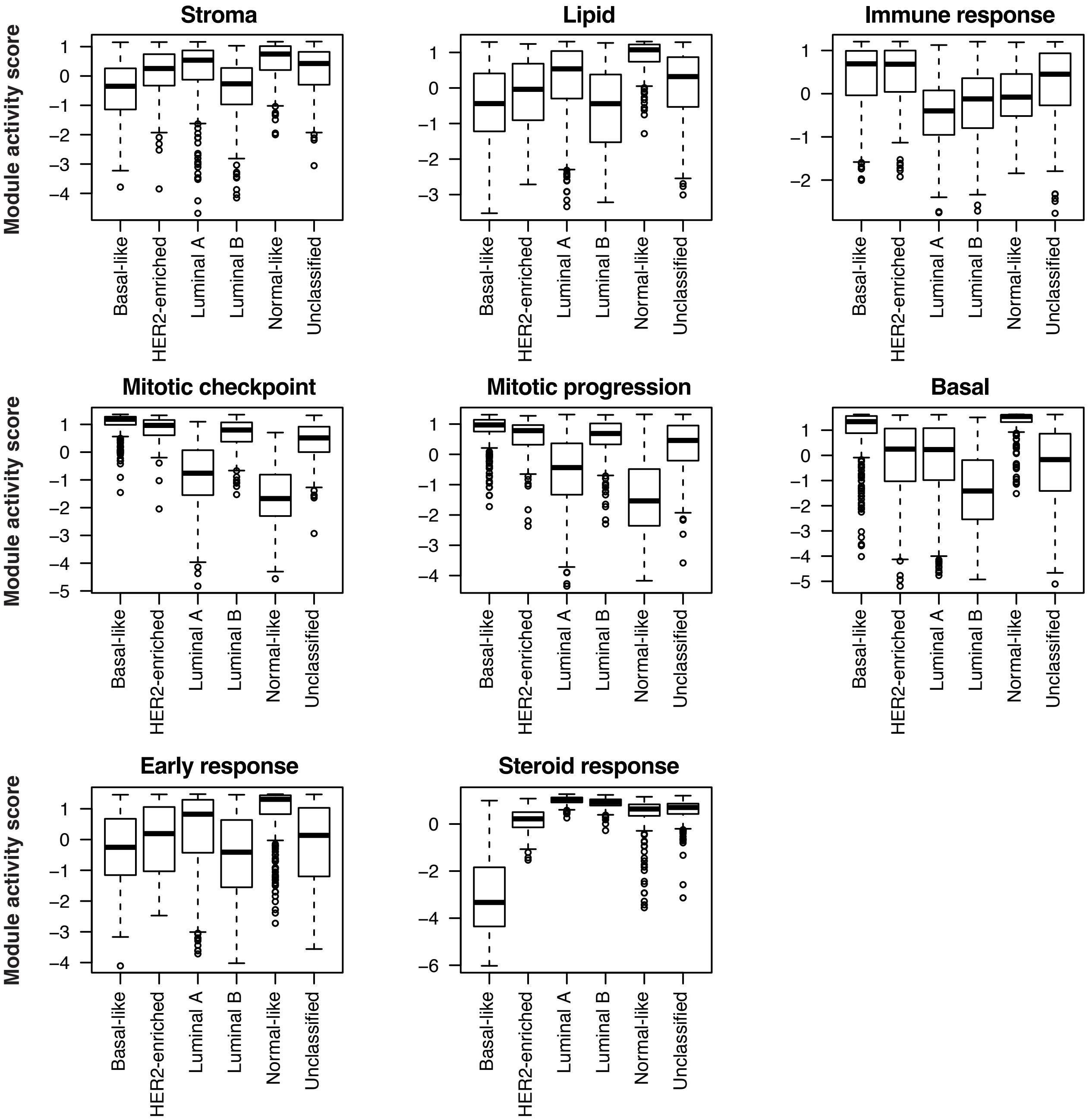

Supplementary Figure 4

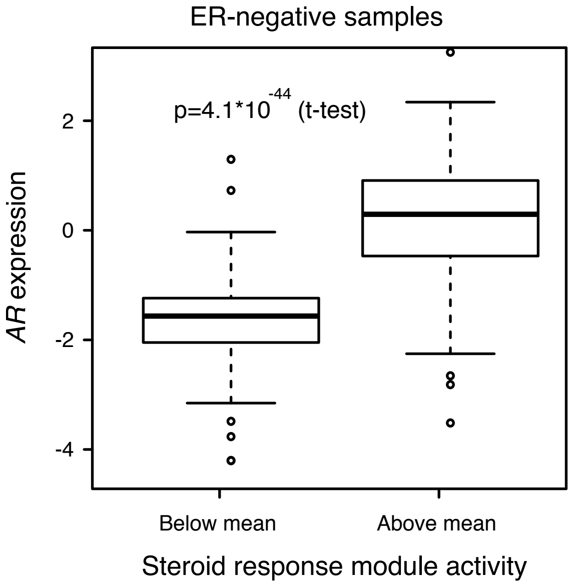

Supplementary Figure 5

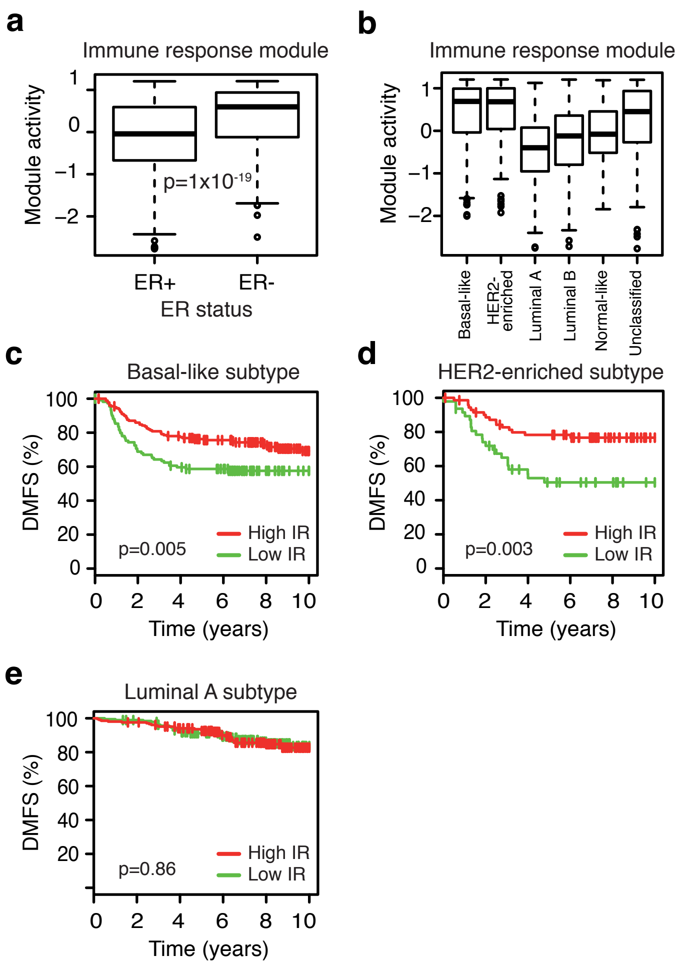

# Supplementary Figure 6

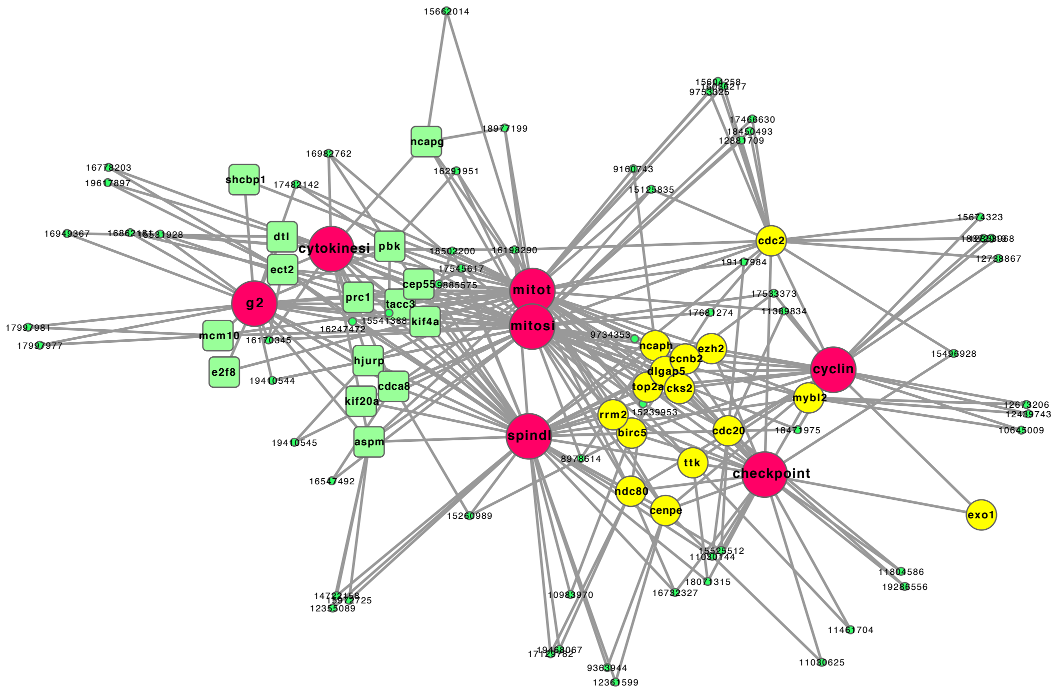

Supplementary Figure 7

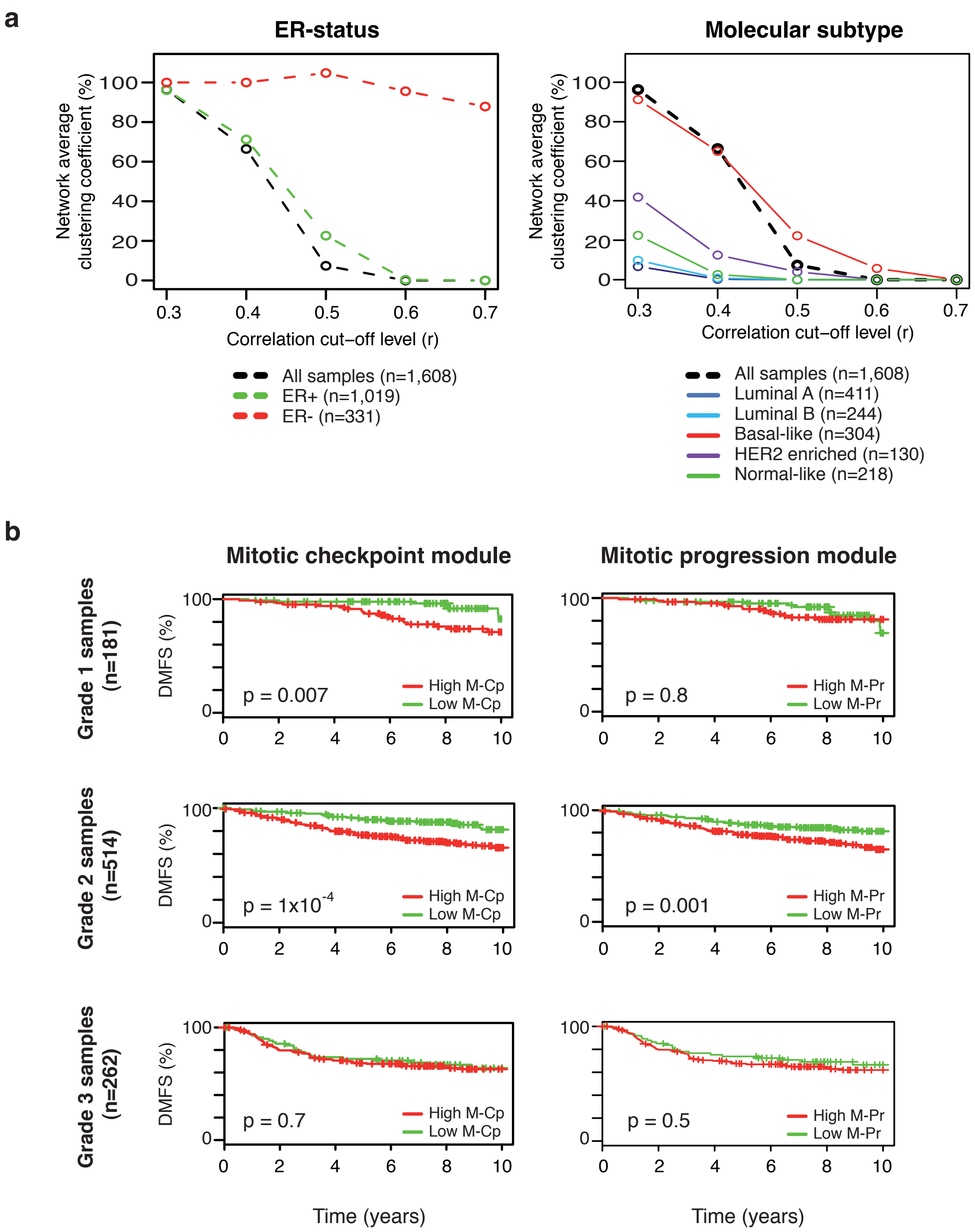

Supplementary Figure 8

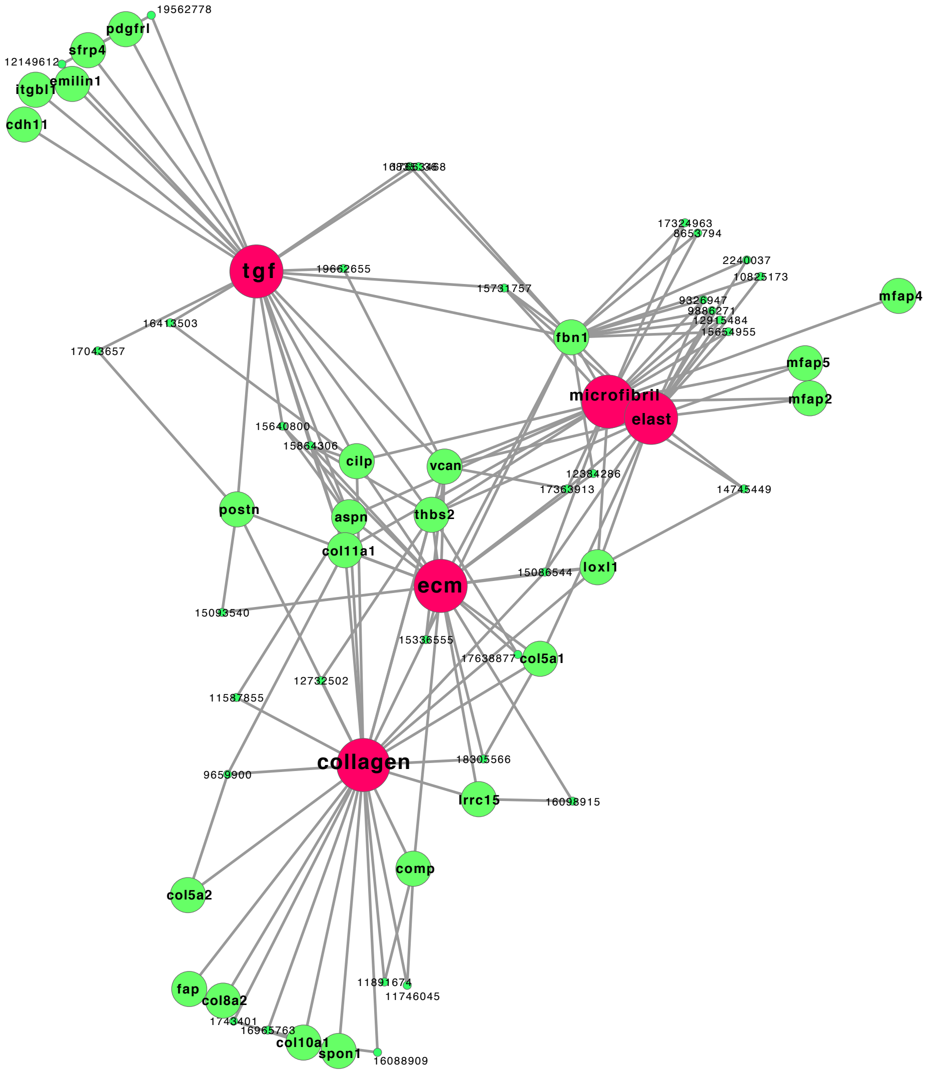

Supplementary Figure 9

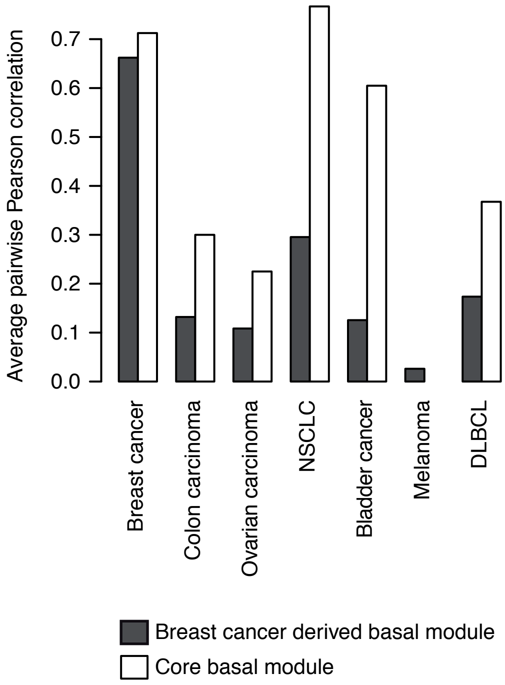

Supplement: Additional file 3 — Supplementary figures. A pdf file containing all supplementary figures with legends (Figure S1-S9). [file bcr3236-S3.PDF]
